# Laparoscopic Sleeve Gastrectomy in Monozygotic Twins: Report of the First Case

**DOI:** 10.7759/cureus.35665

**Published:** 2023-03-01

**Authors:** Elnur Huseynov, Gulcan Coban, Vusal Aliyev

**Affiliations:** 1 General and Obesity Surgery, Avrupa Safak Hospital, Istanbul, TUR; 2 General Surgery, Avrupa Safak Hospital, Istanbul, TUR; 3 General Surgery, Bogazici Academy for Clinical Sciences, Istanbul, TUR

**Keywords:** laparoscopy, siblings, obesity, sleeve gastrectomy, monozygotic twins

## Abstract

Obesity is an epidemic disease that is believed to link to other pathologies as well as life-threatening cardiovascular pathology. Here we report a case of monozygotic twins who successfully lost weight following laparoscopic sleeve gastrectomy at the end of the 18-month follow-up period. We aimed to determine the factors affecting the weight loss outcome after sleeve gastrectomy in monozygotic twins. The twins' initial BMIs were 37.1 kg/m2 and 40.2 kg/m2, respectively. Twin A's excess weight loss was 48.4%, 61.3%, 80.6%, 96.8%, and 112.9% at three, six, nine, 12 and 18 months, while Twin B's in the third, sixth, ninth, 12th and 18th months was 23.1%, 41%, 51.3%, 61.5% and 71.8%.

On the third, sixth, ninth, 12th, and 18th months of Twin A, the total weight loss was 15.8%, 20%, 26.3%, 31.6%, and 36.8%. In Twin B in the third, sixth, ninth, 12th, and 18th months, it was 8.7%, 15.5%, 19.4%, 23.3%, and 27.2%. When the twins were compared in terms of excess weight loss and total weight loss at 18 months, Twin A was more successful than Twin B. Especially at this point, Twin B's having a child (three years old) at a young age, her low compliance with the recommendations in the post-operative period and her difficulty in changing her lifestyle, environmental factors are as important as hereditary factors in achieving weight loss and a healthy body mass index (BMI) range.

## Introduction

Obesity is an epidemic disease characterized by excessive fat accumulation in the body, which adversely affects energy intake, energy expenditure, metabolic efficiency and reward pathways, and impairs health. When it comes to success in obesity treatment, it is among the goals to reduce the morbidity and mortality risks related to obesity, to provide the individual with adequate and balanced nutrition habits and to increase their quality of life. However, long-term success of behavioral interventions and pharmacotherapy targeting the mechanisms underlying obesity is known to be limited [1-3]. Especially in morbidly obese patients, the fact that bariatric surgery is the only treatment that provides an average of more than 15% weight loss in the last 10 years suggests that surgical treatment may be effective for definitive success when diet, exercise and behavioral changes are essential [3,4]. It is known that many factors are effective for weight loss after surgery, especially genetic and environmental factors that interact in a complicated way. With this case report, we aimed to determine the factors affecting the weight loss outcome after sleeve gastrectomy in monozygotic twins (MZ) and to shed light on similar studies with a larger cohort in the future, and we created this case report according to the Surgical CAse REport (SCARE) criteria [5].

## Case presentation

Thirty-six-year-old Turkish female monozygotic twins who had exceeded weight for a long time were admitted to our clinic for evaluation for bariatric surgery. Before the operation, Twin A was 95 kg and her body mass index (BMI) was 37.1 kg/m2. She had ulcerative colitis and was in remission during preoperative investigations. She had previously used mesalazine and azathioprine. A gastroenterologist consultation was made before the operation and there were no contraindications for the bariatric surgery. She had no history of smoking and alcohol use. Twin B was 103 kg and BMI was 40.3 kg/m. She had hypertension and pituitary adenoma in her medical history. She did not regularly use any medication or supplements. She was married and a mother of one child and was engaged in a textile business similar to Twin A. There was no smoking or alcohol use in her social history (Table 1). 

**Table 1:** Summary of characteristics of the twins. BS (Before Surgery), BMI (Body Mass Index), OGD (esophagogastroduodenoscopy)

	Twin A	Twin B
Age at BS, years	37	37
Past medical history	Ulcerative colitis	Hypophysis adenoma, Arterial hypertension
Drug use	Mesalazine, Azathioprine	None
Social history	Non-smoker, Drinks alcohol socially, Works as a career (full time), Lives with family	Non-smoker, Non-alcoholic, Works as a career (full time), Lives with partner and has 1 child
Initial BMI, kg/m^2^	37.1	40.2
OGD	Normal	Normal
Presence of H. pylori in specimen	None	None
Attended Bariatric Group Education Session	Yes	Yes
Post-operative Complications	None	None
Length of hospital stay (days)	2	2

Both twins were evaluated preoperatively by the same professional bariatric surgery team. A two-week preoperative liver reduction diet was given to both. They had surgery a few days apart. In both patients, the abdomen was entered with a standard visible 12 mm port. The liver was retracted with a 5 mm Nathanson liver retractor under the xiphoid. The abdomen was entered with 15 mm ports from the right midclavicular midline lateral to the umbilicus and 12 mm from the left midclavicular midline. The stomach was separated from the omentum with an energy device LigaSure™ (LS) (Covidien, Boulder, CO, USA). Sleeve gastrectomy was performed with a 36 Fr (12 mm) bougie and EndoGIA 60 mm from 4 cm proximal to the pylorus. The resected part of the stomach was removed from the right 15 mm port out of the abdomen. A 12 mm Jackson-Pratt drain was placed adjacent to the stomach. No complications were observed in either patient after surgery, and the abdominal drains were removed two days later and they were discharged without any problems. After the operation, the patients were followed up regularly for up to 18 months.

Weight loss and other results

**Figure 1:**
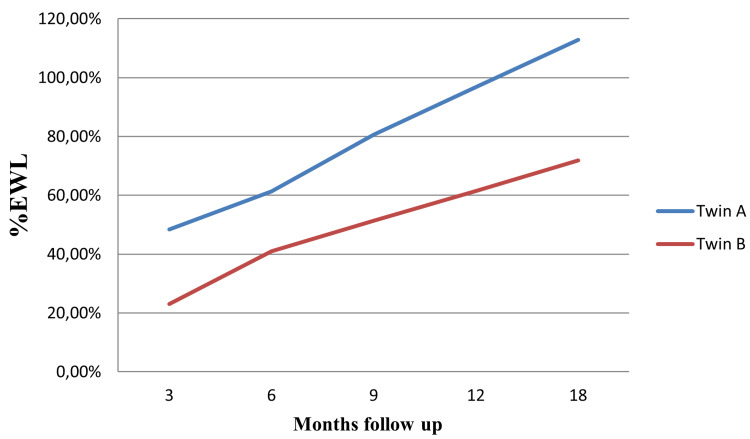
%EWL monthly exchange EWL (Excess Weight Loss)

**Figure 2:**
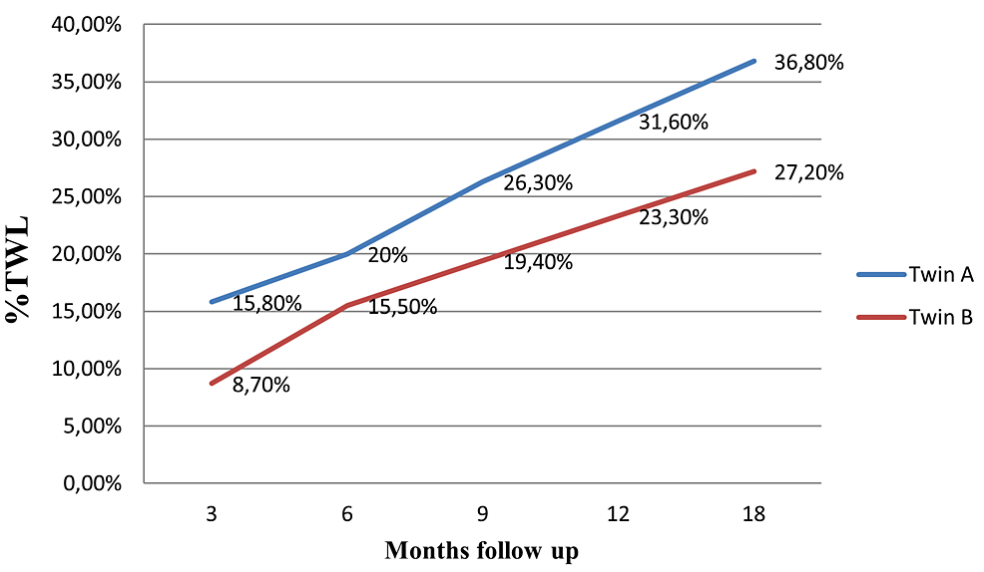
%TWL monthly exchange TWL (Total Weight Loss)

Twin A's excess weight loss (%EWL) was 48.4%, 61.3%, 80.6%, 96.8%, and 112.9% at three, six, nine, 12 and 18 months, while Twin B's in the third, sixth, ninth, 12th and 18th months was 23.1%, 41%, 51.3%, 61.5% and 71.8% (Figure 1). At the third, sixth, ninth, 12th, and 18th months of Twin A, total weight loss (%TWL) was 15.8%, 20%, 26.3%, 31.6%, and 36.8%. In Twin B in the third, sixth, ninth, 12th, and 18th months, the %TWL was 8.7%, 15.5%, 19.4%, 23.3%, and 27.2% (Figure 2). 

**Figure 3:**
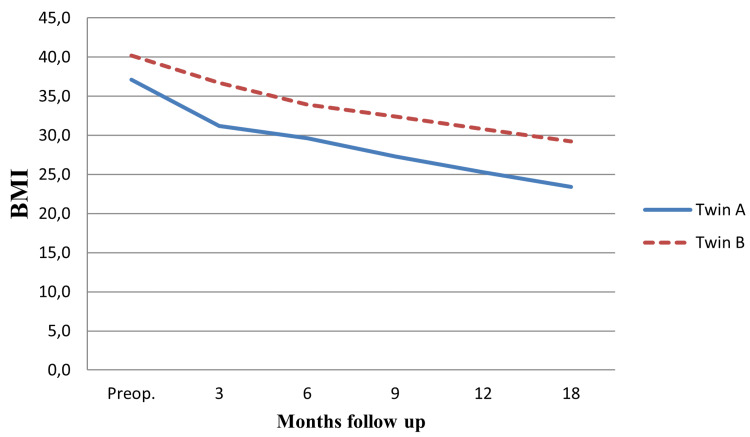
BMI Months follow up BMI (Body Mass Index)

When the MZ twins were compared in terms of %EWL and %TWL at 18 months, Twin A was more successful than Twin B. Especially at this point, Twin B's having a child (three years old) at a young age, her low compliance with the recommendations in the postoperative period and her difficulty in changing her lifestyle, environmental factors are as important as hereditary factors in achieving weight loss and a healthy BMI range (Figure 3).

Additionally, we observed a significant decrease in liver enzymes in both patients when pre- and postoperative blood parameters were taken into account (Table 2). Again, we observed a high improvement in the lipid profile for weight loss in both twins. However, there was no significant difference between the monozygotic twins in terms of changes in blood parameters. In addition, no nutritional deficiency was observed in either patient during or after the follow-up period.

**Table 2:** Preoperative and postoperative laboratory values for twins HbA1C (Hemoglobin A1c), TG (Triglyceride), LDL (Low Density Lipoprotein), ALT (Alanine Transaminase), AST (Aspartate Aminotransferase)

	PREOPERATIVE		POSTOPERATIVE (18 month)	
	Twin A	Twin B	Twin A	Twin B
HbA1C	5.68%	5.60%	5.20%	5.20%
Fasting Blood Sugar	96	103	82	91
Total Cholesterol	278	326	220	289
TG	209	270	128.6	149
HDL	88.9	75.9	69.8	76
LDL	159.9	196.1	124.5	183
ALT	45	97	9	15
AST	40	64	12	14

## Discussion

Genetic factors have an essential role in determination and protection of body weight. The results of studies examining the role of genetics in determining weight loss outcomes following bariatric surgery (BS) show that genetic and environmental factors interact in a complex way in weight maintenance and response to surgery. Some studies have emphasized that genetic contributions to weight and obesity are quite strong and probably have a greater influence than environmental influences [6-8]. A previously published study showed that BMI is strongly congruent between monozygotic twin pairs [9].

However, we present the first report examining the response of monozygotic twins to BS with blood parameters in addition to BMI, %EWL, %TWL rates after sleeve gastrectomy, which is the gold standard of bariatric surgical procedures. At 18 months follow-up, we observed the potential impact of social support and postoperative management on postoperative weight loss, although genetics had a strong influence on weight loss and maintenance, similar to previous reports in the literature [10].

In another similar study [11] investigating the effect of hereditary factors on weight loss following surgical treatment of obesity, non-adjustable gastric banding (LAGB) was applied to MZ twins. A similarity was observed in BMI, cholesterol, and triglycerides after postoperative follow-up. As a contribution, we observed a similar significant change, especially in blood lipids, in response to BS in monozygotic twins in this case report.

In a similar case report [10], the potential for postoperative genetic contribution was consistent in four monozygotic twins who underwent Roux-en-Y Gastric Bypass (RYGB). Twins undergoing RYGB exhibited nearly identical weight loss results after surgery, whereas twins undergoing adjustable gastric banding had quite different weight loss results. This illustrates the influence of postoperative management and the influence of different social situations, although genes are known to have a strong influence on weight loss. On the other hand, we contribute to the literature with a different operation procedure by reporting the weight loss results in MZ twins to which we applied sleeve gastrectomy.

## Conclusions

This report is the first case of sleeve gastrectomy in monozygotic twins. Successful weight loss was achieved at the end of the 18-month follow-up period. In conclusion, we emphasize that although genes have a strong influence on weight loss, the impact of postoperative management and the importance of environmental factors should not be overlooked. In addition, we report that future studies with larger cohorts are needed.
